# Supplementary material for: Deletion of wfs1 Impairs Oligodendrocyte Precursor Cells Dorsal Distribution and Myelination Through the wfs1-hmgcs1 Axis in Zebrafish
Source: Biology (Basel). 2026 Aug 16;15(16):1402. doi: 10.3390/biology15161402 (PMC13510151; doi:10.3390/biology15161402)
Supplement: Supplementary file 1 [file biology-15-01402-s001.zip › File S1-Transcriptome sequencing data and differentially expressed genes (DEGs) in wfs1-- zebrafish at 5 dpf/2.KEGG/wfs1KOvsWT/GSEA/wfs1KO/ABC_TRANSPORTERS/ABC_TRANSPORTERS(DRE02010).html]

Details for gene set ABC\_TRANSPORTERS(DRE02010)[GSEA]

|  || Dataset | deg.sample |
| Phenotype | sample.cls |
| Upregulated in class | wfs1KO |
| GeneSet | ABC\_TRANSPORTERS(DRE02010) |
| Enrichment Score (ES) | 0.4125048 |
| Normalized Enrichment Score (NES) | 1.4081062 |
| Nominal p-value | 0.058939096 |
| FDR q-value | 0.2097046 |
| FWER p-Value | 0.875 |
Table: GSEA Results Summary

  

Fig 1: Enrichment plot: ABC\_TRANSPORTERS(DRE02010)      
 Profile of the Running ES Score & Positions of GeneSet Members on the Rank Ordered List

  

| PROBE | DESCRIPTION (from dataset) | GENE SYMBOL | GENE\_TITLE | RANK IN GENE LIST | RANK METRIC SCORE | RUNNING ES | CORE ENRICHMENT || 1 | 406803\_1 | abcd3a |  |  | 478 | 3.059 | 0.0573 | Yes |
| 2 | 368620\_1 | abcc4 |  |  | 626 | 2.732 | 0.1172 | Yes |
| 3 | 570453\_1 | abcg2b |  |  | 1483 | 1.662 | 0.1296 | Yes |
| 4 | 406803\_2 | abcd3a |  |  | 1813 | 1.458 | 0.1537 | Yes |
| 5 | 100002917 | abcc6b.2 |  |  | 1994 | 1.370 | 0.1805 | Yes |
| 6 | 570148\_1 | abcb9 |  |  | 2028 | 1.357 | 0.2115 | Yes |
| 7 | 571189 | abcb11b |  |  | 2122 | 1.316 | 0.2397 | Yes |
| 8 | 797269 | abcb11a |  |  | 2162 | 1.301 | 0.2692 | Yes |
| 9 | 100537809 | LOC100537809 |  |  | 2214 | 1.282 | 0.2979 | Yes |
| 10 | 735310 | abcg2d |  |  | 2500 | 1.173 | 0.3167 | Yes |
| 11 | 393561\_1 | abcc2 |  |  | 2644 | 1.130 | 0.3389 | Yes |
| 12 | 100329706 | abcc8b |  |  | 2652 | 1.127 | 0.3653 | Yes |
| 13 | 556826\_1 | si:dkey-57h18.2 |  |  | 3330 | 0.967 | 0.3669 | Yes |
| 14 | 100136868 | abca1b |  |  | 4077 | 0.815 | 0.3627 | Yes |
| 15 | 735312 | abcg2a |  |  | 4126 | 0.806 | 0.3802 | Yes |
| 16 | 558335 | abca12 |  |  | 4379 | 0.765 | 0.3904 | Yes |
| 17 | 553281\_1 | abcc8 |  |  | 4649 | 0.727 | 0.3991 | Yes |
| 18 | 100333043 | tap1 |  |  | 4759 | 0.711 | 0.4125 | Yes |
| 19 | 564067 | abcb6a |  |  | 5492 | 0.635 | 0.4045 | No |
| 20 | 100136865 | abcb4 |  |  | 6569 | 0.531 | 0.3832 | No |
| 21 | 393561 | abcc2 |  |  | 6884 | 0.502 | 0.3852 | No |
| 22 | 100136850 | abcg8 |  |  | 7449 | 0.457 | 0.3783 | No |
| 23 | 566367 | abcd1 |  |  | 7979 | 0.425 | 0.3717 | No |
| 24 | 568482 | abcc12 |  |  | 8134 | 0.417 | 0.3767 | No |
| 25 | 569858 | abcg2c |  |  | 8859 | 0.377 | 0.3629 | No |
| 26 | 557317 | abcg5 |  |  | 8927 | 0.373 | 0.3696 | No |
| 27 | 101883977 | LOC101883977 |  |  | 8956 | 0.371 | 0.3775 | No |
| 28 | 556709\_1 | abcc10 |  |  | 9078 | 0.361 | 0.3822 | No |
| 29 | 368771 | abcb3l1 |  |  | 9531 | 0.332 | 0.3759 | No |
| 30 | 794238 | abcg4b |  |  | 9980 | 0.304 | 0.3690 | No |
| 31 | 561460 | zgc:172302 |  |  | 10324 | 0.283 | 0.3649 | No |
| 32 | 798527 | abcb5 |  |  | 11852 | 0.198 | 0.3216 | No |
| 33 | 570453 | abcg2b |  |  | 12099 | 0.185 | 0.3182 | No |
| 34 | 336563\_2 | wu:fb13g09 |  |  | 12274 | 0.175 | 0.3169 | No |
| 35 | 559093\_2 | abcc13 |  |  | 12343 | 0.172 | 0.3188 | No |
| 36 | 548340 | abcb8 |  |  | 13142 | 0.133 | 0.2969 | No |
| 37 | 561160 | abcc9 |  |  | 13406 | 0.120 | 0.2915 | No |
| 38 | 566555 | abcc6a |  |  | 13467 | 0.117 | 0.2924 | No |
| 39 | 100002010 | abcc1 |  |  | 14081 | 0.088 | 0.2752 | No |
| 40 | 767748 | abcd4 |  |  | 14904 | 0.051 | 0.2506 | No |
| 41 | 110438354 | LOC110438354 |  |  | 14958 | 0.049 | 0.2501 | No |
| 42 | 564842 | abcg4a |  |  | 15160 | 0.039 | 0.2447 | No |
| 43 | 556979 | abcg1 |  |  | 15215 | 0.036 | 0.2438 | No |
| 44 | 336563\_1 | wu:fb13g09 |  |  | 15592 | 0.018 | 0.2324 | No |
| 45 | 100004801 | abcb6b |  |  | 15866 | 0.004 | 0.2239 | No |
| 46 | 564344 | abca3b |  |  | 16228 | -0.009 | 0.2128 | No |
| 47 | 100151075 | abca5 |  |  | 17291 | -0.045 | 0.1805 | No |
| 48 | 323301 | abcc3 |  |  | 18032 | -0.077 | 0.1591 | No |
| 49 | 555506 | abca4b |  |  | 18353 | -0.092 | 0.1512 | No |
| 50 | 100006090 | abca2 |  |  | 19521 | -0.144 | 0.1179 | No |
| 51 | 566515 | abcb7 |  |  | 20021 | -0.167 | 0.1062 | No |
| 52 | 558924 | abca1a |  |  | 20323 | -0.180 | 0.1010 | No |
| 53 | 336147 | abcc5 |  |  | 20324 | -0.180 | 0.1053 | No |
| 54 | 100003744 | abcb10 |  |  | 22682 | -0.307 | 0.0385 | No |
| 55 | 100170781 | abcd3b |  |  | 22699 | -0.307 | 0.0452 | No |
| 56 | 559080 | cftr |  |  | 22825 | -0.315 | 0.0487 | No |
| 57 | 559080\_1 | cftr |  |  | 22866 | -0.317 | 0.0550 | No |
| 58 | 336563 | wu:fb13g09 |  |  | 26696 | -0.598 | -0.0512 | No |
| 59 | 556826 | si:dkey-57h18.2 |  |  | 27336 | -0.653 | -0.0558 | No |
| 60 | 559093 | abcc13 |  |  | 28248 | -0.777 | -0.0661 | No |
| 61 | 368620 | abcc4 |  |  | 30403 | -1.450 | -0.0995 | No |
| 62 | 406803 | abcd3a |  |  | 31390 | -2.624 | -0.0685 | No |
| 63 | 553281 | abcc8 |  |  | 31669 | -3.561 | 0.0069 | No |
Table: GSEA details [plain text format]

  

Fig 2: ABC\_TRANSPORTERS(DRE02010)      
 Blue-Pink O' Gram in the Space of the Analyzed GeneSet

  

Fig 3: ABC\_TRANSPORTERS(DRE02010): Random ES distribution      
 Gene set null distribution of ES for **ABC\_TRANSPORTERS(DRE02010)**
